# Aspects of Area Deprivation Index in Relation to Hippocampal Volume Among Children

**DOI:** 10.1001/jamanetworkopen.2024.16484

**Published:** 2024-06-12

**Authors:** Benson S. Ku, Katrina Aberizk, Cope Feurer, Qingyue Yuan, Benjamin G. Druss, Dilip V. Jeste, Elaine F. Walker

**Affiliations:** 1 Department of Psychiatry and Behavioral Sciences, Emory University School of Medicine, Atlanta, Georgia; 2 Department of Psychology, Emory University, Atlanta, Georgia; 3 Department of Psychiatry, University of Illinois at Chicago; 4 Department of Health Policy and Management, Rollins School of Public Health, Emory University, Atlanta, Georgia; 5 Global Research Network on Social Determinants of Health and Exposomics, La Jolla, California

## Abstract

**Importance:**

Area deprivation index (ADI) has been shown to be associated with reduced hippocampal volume (HV) among youths. The social environment may interact with the association between ADI and HV.

**Objective:**

To investigate which aspects of ADI are uniquely associated with bilateral HV and whether school and family environments have moderating interactions in associations between ADI and HV.

**Design, Setting, and Participants:**

This cross-sectional study used data from the Adolescent Brain and Cognitive Development (ABCD) study. Participants aged 9 and 10 years were recruited from 21 sites in the US between September 2016 and August 2018. Data analysis was performed between March 2023 and April 2024.

**Exposures:**

ADI aspects were derived from participant primary home addresses provided by parents or guardians.

**Main Outcomes and Measures:**

HV was automatically segmented from structural brain images ascertained from magnetic resonance imaging. Multiple generalized linear mixed modeling tested associations between 9 indices of ADI and bilateral HV, with family groups and recruitment sites as random effects. After stepwise backward selection, models were adjusted for individual-level covariates, including age, sex, race and ethnicity, parental education, household income, and estimated intracranial volume.

**Results:**

This study included 10 114 participants aged 9 and 10 years (median [IQR] age, 9.92 [9.33-10.48] years; 5294 male [52.3%]; 200 Asian [2.0%], 1411 Black [14.0%], and 6655 White [65.8%]; 1959 Hispanic [19.4%]). After stepwise backward selection and adjusting for covariates, only the percentage of neighborhood-level single-parent households was associated with right HV (adjusted β per 1-SD increase in single-parent households, −0.03; 95% CI, −0.06 to −0.01; *P* = .01). School environment interacted with neighborhood-level single-parent households in its association with right HV (adjusted β per 1-SD increase in score, 0.02; 95% CI, 0.01 to 0.03; *P* = .003), such that there was an inverse association only among those at a school with the mean environment score (adjusted β per 1% increase in single-parent households, −0.03; 95% CI, −0.05 to −0.01; *P* = .02) and worse (−1 SD score) school environment score (adjusted β per 1% increase in single-parent households, −0.05; 95% CI, −0.09 to −0.01; *P* < .001) but not among those at better (+1 SD score) school environments.

**Conclusions and Relevance:**

In this study, an increased percentage of neighborhood-level single-parent households was associated with reduced right HV among children in schools with the mean or worse but not better environment score. These findings suggest that longitudinal research concerning the association of neighborhood-level characteristics and school environments with hippocampal development may be warranted to better understand complex interactions between various social factors and child neurodevelopment and mental health outcomes.

## Key Points

**Question:**

Which aspects of the area deprivation index are associated with hippocampal volume in children?

**Findings:**

This cross-sectional study of 10 114 children found an inverse association between neighborhood-level single-parent households and right hippocampal volume, which was moderated by a self-reported positive school environment.

**Meaning:**

These findings suggest that school environment may have a moderating interaction in the association of neighborhood single-parent households with hippocampal development in children.

## Teaser

This cross-sectional study investigates the association of area deprivation index measures with bilateral hippocampal volume in children and assesses whether these associations vary by school and family environment.

## Introduction

Prospective studies have repeatedly shown that children and adolescents from disadvantaged backgrounds are at increased risk for developing serious mental illnesses.^1^ It is well-documented that early life stress is associated with impaired social and emotional functioning in children, and decades of research have demonstrated that the duration, severity, timing, and quality of stressful experiences are associated with socioemotional and neurodevelopmental outcomes.^2,3^ Indeed, mild and relatively brief stressful experiences are generally considered challenges with adaptive benefits, including enhanced memory and decision-making. In contrast, severe and prolonged stress is associated with detrimental functional and structural aberrations to neural structures associated with emotional processes, especially the hippocampus.^4^ Perhaps unsurprisingly, increased stress exposure is associated with an increased risk for various psychiatric conditions, and a reduction in hippocampal volume (HV) has been identified as a nonspecific brain morphological characteristic in human psychopathology.^5,6^ Thus, understanding the most relevant factors associated with HV may help guide effective interventions to mitigate reductions in HV and future psychopathology.

Evidence suggests that distinct forms of stress may have differential associations with neurodevelopment and psychiatric illnesses, including psychotic disorders.^7,8,9,10^ The stimulation discrepancy deprivation model theorizes that various structural environmental exposures (ie, deprivation, discrepancy, and stimulation or threat) may be associated with the risk for psychosis through distinct neurobiological mechanisms.^11^ For example, deprivation (defined as emotional neglect and poverty) was inversely associated with HV, whereas threat (defined as physical, emotional, and sexual abuse) did not have an association.^12^ Racial and ethnic inequality and social fragmentation are classified as discrepancy exposures, which describe environments that foster a sense of social exclusion, isolation, or lack of belonging.^13^ Childhood area–level social fragmentation, defined as the proportion of individuals who moved (ie, residential instability) and single-parent households, has been shown to partially explain the association between urban upbringing and nonaffective psychotic disorders even after adjusting for socioeconomic indices.^14^

Area-level social fragmentation has been empirically constructed to measure the disruption of social ties in the community.^15,16^ A 2023 study^17^ showed that childhood exposure to social fragmentation was not associated with social functioning during childhood but was associated with poorer social functioning in adulthood. Authors suggested that those deficits in social functioning observed later in life may be precipitated by neurobiological changes. A 2020 study^18^ examined that possibility, focusing on neurobiological changes germane to the hippocampus and demonstrating inverse associations between the area deprivation index (ADI) and HV among youths. These findings were consistent with results from rodent studies showing that resource scarcity was associated with structural deficits in the hippocampus, including dendritic atrophy and apoptosis.^19,20,21,22,23^ The ADI has been operationalized as a composite score of various neighborhood-level adverse characteristics, including general socioeconomic status (ie, poverty) and social fragmentation (ie, percentage of single-parent households).^14^ Single-parent households have been shown to be associated with financial hardship and poor social networks, which may lead to disruption of social ties or lack of connections between community members.^14,24,25^ Recent findings suggest that indices that are specific to social fragmentation may be particularly relevant in the associations between ADI and aberrant neurodevelopment and subsequent psychopathology.^16,26,27^

Furthermore, studies have shown that enriched family and school environments may buffer against adverse outcomes associated with neighborhood-level deprivation in adolescent mental health,^28,29^ brain structure, and patterns of brain connectivity.^30,31,32^ For example, a more favorable school environment has been associated with decreased connectivity between task-positive and task-negative brain networks (ie, central executive and default mode networks, respectively) after accounting for neighborhood poverty, child intellectual performance, and demographic characteristics.^30^ Critically, lower connectivity between task-positive and task-negative networks is an adaptive reflection of appropriate network segregation and optimization of metabolic resources in the brain.^30^ Prior studies also demonstrated that a high level of family conflict was associated with behavioral problems and smaller cortical areas in various brain regions in children^33^ and that unfavorable home environments (eg, unsupportive parenting, physical abuse, and low socioeconomic status) were associated with reduced HV in children.^7,34^

Coincident with an ongoing trend toward generating composite indices of socioenvironmental stress (eg, ADI and social fragmentation), it is important to clarify the aspects of these indices that are uniquely associated with adverse neurodevelopmental and socioemotional outcomes in youths. Indeed, it is unclear which aspects of ADI are relevant for hippocampal development. Clarifying aspects of the ADI that are associated with HV is important to elucidate early life exposures to stress that are most pertinent to hippocampal development trajectory. This study aimed to clarify which aspects of ADI were uniquely associated with bilateral HV and whether family and school environments had moderating interactions in the association between ADI and HV. In this study, we used the same sample as those used by Taylor et al.^18^ We extended this work in 2 ways: we used stepwise backward elimination to identify relevant neighborhood characteristics in their association with HV, and we assessed moderating interactions of family and school environments in the association between relevant ADI individual measures and HV. Prior literature has shown small effect sizes for the association of neighborhood deprivation indices with HV.^18,35^ Therefore, it would be important to further investigate subgroups of individuals for whom indices of neighborhood deprivation would contribute to HV. We hypothesized that aspects of the ADI that measure social fragmentation would be associated with reduced HV and that school and family environments would have a moderating interaction with this association.

## Methods

### Design

This cross-sectional study used data collected from the Adolescent Brain Cognitive Development (ABCD) study version 4.0, which aimed to investigate the dynamic processes underpinning adolescent brain development at 21 study sites across the US via school-based recruitment of children aged 9 and 10 years between September 2016 and August 2018.^36^ All study procedures were approved by the University of California, San Diego Institutional Review Board. Informed written consent was obtained from the child and parent. This report follows the Strengthening the Reporting of Observational Studies in Epidemiology (STROBE) reporting guideline.

### Participants

This study excluded participants with missing targeted variables, covariates, and geocoded addresses (eTable 1 in Supplement 1). A comparison of sociodemographic characteristics between 10 114 included and 1762 excluded participants is presented in eTable 2 in Supplement 1. Distributions of individual and neighborhood characteristics from included and excluded participants were visually inspected using quantile-quantile and box plots (eFigures 1-3 in Supplement 1).

### Sociodemographic Characteristics

Information on sociodemographic characteristics, including age, biological sex, level of parental education, race and ethnicity, and total combined family income, was collected through self-report and interviews conducted at baseline. Available race categories were American Indian or Alaskan Native, Asian, Black, Native Hawaiian or Pacific Islander, White, and other, which included participants who reported a race that was not included in the list, did not know their race, or did not disclose their race. Ethnicity categories were Hispanic or Latino or not Hispanic or Latino. Race and ethnicity were assessed as part of the baseline demographic characteristics from the ABCD study. This study dichotomized race and ethnicity as done in prior literature^35^ as non-Hispanic White, which served as the reference category, and others, which includes all other races and Hispanic or Latino ethnicity. Parental high school education was defined as having at least 1 of the parents or caregivers obtain a high school diploma.

### Neighborhood Characteristics

For the ABCD study, neighborhood-level characteristics were obtained using primary home addresses of participants obtained at baseline, which were then geocoded to access census tract–level data. Neighborhood characteristics used in this study were obtained from the 2010 American Community Survey 5-year summary estimates (mean annual estimates spanning 2010-2014).

We examined the 9 ADI measures individually rather than aggregating them into a single neighborhood index, as done by Taylor et al.^18^ These indices include the percentage of the population aged 25 years and older with at least a high school diploma, median family income, income disparity (defined by the ratio of low- to high-income households), percentage of households with respondents who were homeowners, percentage of the civilian labor force population aged16 years or older who were unemployed, percentage of families below the poverty level, percentage of the population below 138% of the poverty threshold, percentage of single-parent households, and percentage of occupied housing units without a motor vehicle.^18^ Among these indices, the percentage of single-parent households has been used to measure childhood social fragmentation.^14,17^

### School Environment

Positive school environment was assessed using the school environment subscale from the ABCD School Risk and Protective Factors Survey.^37^ The score was calculated by the sum of six 4-item subscales: availability of extracurricular activities, relationship with teachers, receiving praise for good performance, opportunities to contribute to class activities, school-parent communications, and sense of safety at school.

### Family Environment

The acceptance subscale from the Children’s Report of Parental Behavior Inventory was used to assess positive parenting.^38,39^ This inventory includes five 3-item subscales that measure various aspects of the parent-child relationship, such as showing affection by smiling at the child, discussing the child’s worries, and providing support.

### Imaging Data Acquisition and Processing

All participating sites conducted structural magnetic resonance imaging (MRI) using similar sequences on 3T scanners from Siemens, Phillips, or General Electric, each equipped with a 32-channel head coil. While participants watched a child-appropriate movie of their choice, a 3-dimensional, T1-weighted image with a 1-mm isotropic voxel resolution was acquired.^40,41^ To maintain data quality and minimize motion artifacts, real-time motion detection and correction software were used at Siemens and General Electric sites.^42,43^

The FreeSurfer software suite version 5.3 (Martinos Center for Biomedical Imaging) was used to perform automated segmentation of subcortical structures, including the hippocampus.^41^ HV measures were extracted using a Desikan atlas with 34 parcels in each hemisphere based on gyral and sulcal patterns. The reliability of HV measures was demonstrated across MRI scanners used in this study.^40,41^ Scans from participants rated as unusable were excluded from the released dataset. A sensitivity analysis for quality control of imaging data was conducted. In this sensitivity analysis, we included only individuals who met the criteria for “T1w data recommended for inclusion,” which was a subset of 9081 individuals who passed quality control for FreeSurfer and T1 series and did not have missing quality control data (imgincl_t1w_include = 1).^41,44^

### Statistical Analysis

Distributions of each component of ADI, covariates used in our model, outcome variables (eg, HV), and their associations were visually inspected (eFigures 4 and 5 in Supplement 1). Multiple generalized linear mixed modeling tested associations between 9 indices of ADI and bilateral HV using lme4 and lmerTest packages.^45,46^ Family groups and recruitment sites were treated as random effects because individuals were clustered within families and recruitment sites.

First, all 9 indices of ADI were included in the model. We used the stepwise backward selection process to identify individual ADI items that may uniquely contribute to the variance in HV. As done in prior literature, we applied the standard significance level for hypothesis testing (2-sided α = .05).^47,48^ We then adjusted any remaining items from ADI for 6 individual-level covariates, including age, male sex, White non-Hispanic race and ethnicity, parental high school education, household income, and estimated intracranial volume, shown in previous literature to be associated with HV.^49,50,51^ Variance inflation factors of less than 5 ruled out multicollinearity in the adjusted model (eTable 3 in Supplement 1).^52,53^

For ADI indices that remained associated with HV after adjusting for covariates, we further investigated potential moderating interactions of family and school environments. In 2 separate models, interaction terms were included to test their potential moderating interactions. To better interpret significant interaction terms, we calculated simple slopes to examine associations between the remaining significant ADI indices and HV based on the −1 SD, mean, and +1 SD of the factor using the interactions package.^54^ The final model selection of independent variables and interaction terms were internally validated by bootstrapping with 5000 resamples. All statistical analyses were performed using R statistical software version 4.2.1 (R Project for Statistical Computing). Data were analyzed from March 2023 to April 2024.

## Results

### Descriptive Statistics

This study included 10 114 participants aged 9 to 10 years (median [IQR] age, 9.92 [9.33-10.48] years; 5294 male [52.3%]; 200 Asian [2.0%], 1411 Black [14.0%], and 6655 White [65.8%]; 1959 Hispanic [19.4%])(Table 1**)**. Male participants, non-Hispanic White participants, and those with high parental education showed greater bilateral HV compared with their respective counterparts (eFigure 6 in Supplement 1). Pairwise correlation and scatter plots of independent variables (ADI and covariates) and bilateral HV area are shown in eFigures 7 and 8 in Supplement 1, and family environment but not school environment was correlated with indices of ADIs (eFigure 9 in Supplement 1).

**Table 1.** Baseline Sociodemographic Characteristics

Characteristic	Participants, No. (%) (N = 10 114)
Age, median (IQR), y	9.92 (9.33-10.48)
Sex	
Male	5294 (52.3)
Female	4820 (47.6)
Race	
American Indian or Alaskan Native	50 (0.5)
Asian	200 (2.0)
Black	1411 (14.0)
Native Hawaiian or Pacific Islander	10 (0.1)
White	6655 (65.8)
White non-Hispanic	6324 (62.5)
Other^a^	525 (5.2)
≥2 Races	1263 (12.5)
Hispanic or Latino	1959 (19.4)
Parents with a high school diploma or greater	9718 (96.1)
Total combined family income bracket, median (IQR)	8.00 (6.00-9.00)
Neighborhood-level deprivation index, median (IQR)	
High school diploma, %	92.55 (85.10-96.26)
Annual household income, median (IQR), $	73 333.00 (51 584.75-97 909.50)
Income disparity	2.01 (1.24-2.88)
Homeowners, %	71.20 (52.14-83.86)
Unemployed, %	7.37 (4.94-10.95)
Below poverty line, %	6.89 (3.13-14.66)
Below 138% of poverty line, %	15.75 (8.97-28.59)
Single parents, %	13.91 (8.74-22.70)
No car, %	4.79 (2.13-10.41)
Volume, median (IQR), mm^3^	
Intracranial	1 489 639.50 (1 393 115.50-1 588 378.25)
Left hippocampal	4015.45 (3757.40-4287.00)
Right hippocampal	4137.85 (3866.93-4425.38)
Positive school environment, median (IQR)	20.00 (18.00-22.00)
Positive family environment, median (IQR)	3.00 (2.60-3.00)

^a^ Others included participants who reported a race that was not included in the list, did not know their race, or did not disclose.

### Generalized Linear Mixed Models for Association With HV

After stepwise backward elimination, 3 ADI measures remained associated with right HV. These were percentage of single-parent households (unadjusted β per 1-SD increase, −0.07; 95% CI, −0.10 to −0.04; *P* < .001), mean household income (unadjusted β per 1-SD increase, 0.06; 95% CI, 0.03 to 0.09; *P* < .001), and percentage of the civilian labor force population aged 16 years or older who were unemployed (unadjusted β per 1-SD increase, −0.08; 95% CI, −0.10 to −0.05; *P* < .001) (eTable 4 in Supplement 1).

We then adjusted for 6 individual-level covariates: biological sex, age, White non-Hispanic race and ethnicity, parental high school education, family income, and estimated intracranial volume. Only the neighborhood-level percentage of single-parent households remained associated with reduced right HV (adjusted β per 1-SD increase, −0.03; 95% CI, −0.06 to −0.01; *P* = .01) (Table 2).

**Table 2.** Multivariable Models for Bilateral HV

Variable	Adjusted model		Bootstrapped model	
	β (95% CI)^a^	*P* value	β (95% CI)^a^	*P* value
**Left HV** ^b^				
Individual level				
Age, y	0.02 (0.00 to 0.04)	.19	0.02 (0.00 to 0.04)	.006
Male sex	0.09 (0.05 to 0.13)	<.001	0.09 (0.06 to 0.13)	<.001
White non-Hispanic	0.10 (0.06 to 0.14)	.009	0.10 (0.06 to 0.14)	<.001
Parental high school education	0.03 (−0.05 to 0.11)	.45	0.03 (−0.05 to 0.11)	.23
Total combined family income bracket	0.05 (0.03 to 0.07)	<.001	0.05 (0.03 to 0.07)	<.001
Intracranial volume, mm^3^	0.66 (0.64 to 0.68)	<.001	0.66 (0.64 to 0.67)	<.001
Neighborhood level				
Median family income, $	−0.01 (−0.03 to 0.01)	.23	−0.02 (−0.03 to 0.00)	.12
Proportion with high school diploma, %	0.01 (−0.01 to 0.03)	.62	0.01 (−0.01 to 0.03)	.31
Proportion unemployed, %	−0.01 (−0.03 to 0.01)	.31	−0.01 (−0.05 to 0.03)	.15
Proportion single parents, %	−0.02 (−0.04 to 0.00)	.11	−0.02 (−0.04 to 0.00)	.06
**Right HV** ^c^				
Individual level				
Age, y	0.01 (0.00 to 0.02)	.19	0.01 (−0.02 to 0.03)	.09
Male sex	0.07 (0.06 to 0.09)	<.001	0.07 (−0.02 to 0.02)	<.001
White non-Hispanic	0.05 (0.01 to 0.09)	.009	0.05 (−0.02 to 0.01)	.002
Parental high school education	−0.03 (−0.12 to 0.05)	.45	−0.04 (−0.02 to 0.00)	.21
Total combined family income bracket	0.04 (0.03 to 0.05)	<.001	0.04 (−0.02 to 0.01)	<.001
Intracranial volume, mm^3^	0.63 (0.61 to 0.65)	<.001	0.63 (−0.02 to 0.02)	<.001
Neighborhood level				
Median family income, $	<.01 (−0.02 to 0.03)	.96	<.01 (−0.02 to 0.03)	.48
Proportion unemployed, %	−0.02 (−0.04 to 0.01)	.18	−0.02 (−0.04 to 0.01)	.09
Proportion single parents, %	−0.03 (−0.05 to −0.01)	.02	−0.03 (−0.06 to −0.01)	.01

Abbreviation: HV, hippocampal volume.

^a^ All continuous variables were *z* scored, so all comparisons for continuous variables are per 1-SD increase.

^b^ In adjusted and bootstrapped models, the intraclass correlation of family groups was 0.41, intraclass correlation of sites was 0.03, and pseudo-*R*^2^ was 0.71.

^c^ In adjusted and bootstrapped models, the intraclass correlation of family groups was 0.38, intraclass correlation of sites was 0.02, and pseudo-*R*^2^ was 0.66.

Before adjustment for covariates, 4 neighborhood-level characteristics remained associated with left HV: percentage of population aged 25 years or older with at least a high school diploma (β per 1-SD increase, 0.05; 95% CI, 0.02 to 0.08; *P* < .001), median family income (β per 1-SD increase, 0.04; 95% CI, 0.01 to 0.07; *P* = .02), percentage of the civilian labor force population aged 16 years or older who were unemployed (β per 1-SD increase, −0.07; 95% CI, −0.10 to −0.04; *P* < .001), and percentage of single-parent households (−0.06; 95% CI, −0.09 to −0.03; *P* < .001). However, after adjusting for 6 individual-level covariates, no ADI indices were associated with left HV (eTable 4 in Supplement 1).

### Moderating Interaction of Family and School Environment

The interaction term percentage of single-parent households by school environment (adjusted β per 1-SD increase, 0.02; 95% CI, 0.01 to 0.03; *P* = .003) was associated with right HV (Table 3). However, the interaction term by family environment was not associated with right HV (adjusted β per 1-SD increase, <−0.01; 95% CI, −0.02 to 0.01; *P* = .28) (Table 4). Simple slopes of the significant interaction term are shown in the Figure. There was no association between neighborhood percentage of single-parent households and right HV in better (+1 SD) school environments (adjusted β per 1-SD increase, −0.01; 95% CI, −0.05 to 0.03; *P* = .44). However, there was a negative association in worse (−1 SD) school environments (adjusted β per 1-SD increase, −0.05; 95% CI, −0.09 to −0.01; *P* < .001) and in schools with the mean environment score (adjusted β per 1-SD increase, −0.03; 95% CI, −0.05 to −0.01; *P* = .02). A sensitivity analysis using only structural images that passed strict quality control in the original ABCD dataset demonstrated similar results (eTables 5 and 6 in Supplement 1).

**Table 3.** Interaction Between Neighborhood Single-Parent Households and School Environment for Right Hippocampal Volume

Variable	Interaction model with school environment		Bootstrap interaction model with school environment	
	β (95% CI)^a^^,^^b^	*P* value	β (95% CI)^a^^,^^b^	*P* value
Individual level				
Age, y	0.02 (−0.01 to 0.04)	.19	0.02 (−0.01 to 0.04)	.09
Male sex	0.07 (0.04 to 0.11)	<.001	0.07 (0.04 to 0.11)	<.001
White non-Hispanic	0.05 (0.01 to 0.09)	.008	0.05 (0.01 to 0.10)	<.001
Parental high school education	−0.03 (−0.12 to 0.06)	.49	−0.03 (−0.12 to 0.05)	.25
Total combined family income bracket	0.02 (0.01 to 0.03)	<.001	0.02 (0.01 to 0.03)	<.001
Intracranial volume, mm^3^	0.63 (0.61 to 0.65)	<.001	0.63 (0.61 to 0.65)	<.001
Positive school environment	0.01 (−0.01 to 0.02)	.11	0.01 (−0.01 to 0.02)	.22
Neighborhood level				
Median family income, $	<0.01 (−0.02 to 0.03)	.94	<0.01 (−0.02 to 0.03)	.47
Proportion unemployed, %	−0.01 (−0.04 to 0.01)	.20	−0.02 (−0.04 to 0.01)	.10
Proportion single parents, %	−0.03 (−0.06 to 0.00)	.001	−0.03 (−0.06 to 0.00)	.01
Interaction term: percentage single parents by school environment	0.02 (0.01 to 0.03)	.007	0.02 (0.01 to 0.03)	.003

^a^ All continuous variables were *z* scored, so all comparisons for continuous variables are per 1-SD increase.

^b^ In adjusted and bootstrapped models, the intraclass correlation of family groups was 0.38, intraclass correlation of sites was 0.02, and pseudo-*R*^2^ was 0.66.

**Table 4.** Interaction Between Neighborhood Single-Parent Households and Family Environment for Right Hippocampal Volume

Variable	Interaction model with family environment		Bootstrap interaction model with family environment	
	β (95% CI)^a^^,^^b^	*P* value	β (95% CI)^a^^,^^b^	*P* value
Individual level				
Age, y	0.02 (−0.01 to 0.04)	.20	0.02 (−0.01 to 0.04)	.10
Male sex	0.07 (0.04 to 0.11)	<.001	0.07 (0.04 to 0.11)	<.001
White non-Hispanic	0.05 (0.01 to 0.09)	.009	0.05 (0.01 to 0.10)	<.001
Parental high school education	−0.03 (−0.12 to 0.05)	.45	−0.03 (−0.12 to 0.05)	.22
Total combined family income bracket	0.02 (0.01 to 0.03)	<.001	0.02 (0.01 to 0.03)	<.001
Intracranial volume, mm^3^	0.63 (0.61 to 0.65)	<.001	0.63 (0.61 to 0.65)	<.001
Positive family environment	0.01 (−0.01 to 0.02)	.41	0.01 (−0.01 to 0.02)	.20
Neighborhood level				
Median family income, $	<0.01 (−0.02 to 0.03)	.97	<0.01 (−0.02 to 0.03)	.49
Proportion unemployed, %	−0.02 (−0.04 to 0.01)	.18	−0.02 (−0.04 to 0.01)	.09
Proportion single parents, %	−0.03 (−0.06 to 0.00)	.02	−0.03 (−0.06 to 0.00)	.01
Interaction term: percentage single parents by family environment	<−0.01 (−0.02 to 0.01)	.54	<−0.01 (−0.02 to 0.01)	.28

^a^ All continuous variables were *z* scored, so all comparisons for continuous variables are per 1-SD increase.

^b^ In adjusted and bootstrapped models, the intraclass correlation of family groups was 0.38, intraclass correlation of sites was 0.02, and pseudo-*R*^2^ was 0.66.

**Figure.**
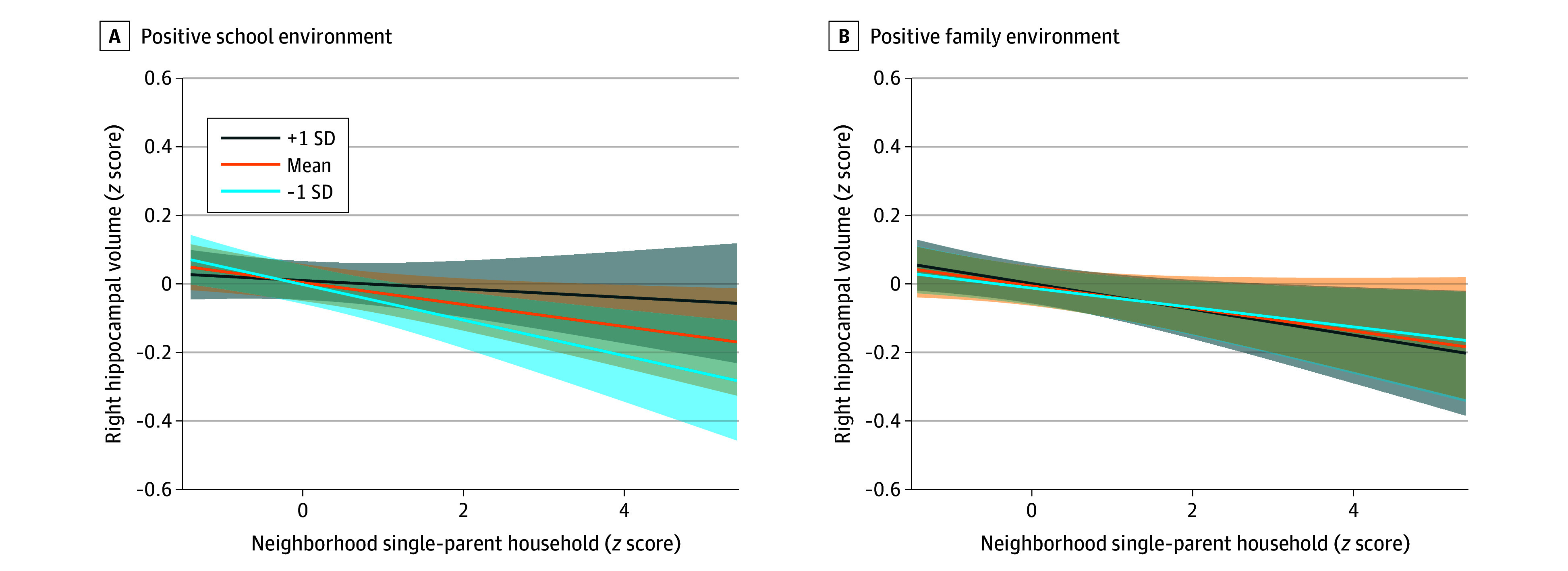
Plots of Interaction Terms for Right Hippocampal Volume Both models were adjusted for 1) fixed effects, including age, male sex, White non-Hispanic race and ethnicity, parental high school education, total combined family income, intracranial volume, neighborhood-level characteristics (median family income, percentage of the civilian labor force population aged ≥16 years who were unemployed, and percentage of single-parent households), and positive school environment, and 2) random effects, including family groups and sites. The interaction term school environment by neighborhood single-parent households was associated with right hippocampal volume; therefore, simple slopes were calculated to interpret the significant interaction term by worse school environment (−1 SD), mean school environment, and better school environment (+1 SD).

## Discussion

This cross-sectional study found that a greater percentage of neighborhood-level single-parent households was associated with reduced right HV among children after accounting for individual-level covariates (eg, socioeconomic status, age, and biological sex). Consistent with prior reports, ADI was associated with only the right HV.^18^ It has been suggested that the right HV is more vulnerable to early life stress than the left HV.^55^ Evidence from animal models suggests that early life environmental enrichment is associated with increased right but not left hippocampus short- and long-term potentiation^56^ and volumetric asymmetry.^57^ Neighborhood-level single-parent households have been proposed as a proxy measure of disrupted family structures. A large, nationally representative study found an inverse association between the proportion of neighborhood single-parent households and upward economic mobility.^58^ These findings suggest that children raised in communities with 2-parent families were significantly more likely to overcome poverty as adults. It has been theorized that family structure plays a crucial role in supporting children’s development, and a greater proportion of 2-parent households in the community would putatively offer more stability and intact social networks for youths.^24^ Conversely, single-parent households have been shown to have poorer social networks compared with 2-parent households, and these households may have less time to engage in community social activities.^24,25^ A greater proportion of single-parent households may be associated with reduced social capital in the community,^24^ which may, in turn, be associated with downstream outcomes for social interactions among peers. Indeed, the percentage of single-parent households has been used to empirically measure area-level social fragmentation, which is associated with school maladaptation during childhood^1^ and subsequent risk for psychosis.^14,59^

Moreover, we found that school environment had a moderating interaction with the inverse association between neighborhood single-parent households and right HV, such that there was an association only among individuals in worse or mean school environments but not among those in better school environments. Our finding suggests that better school environments may buffer against the association of adverse social environments with reduced HV among children. This finding builds upon prior research suggesting the protective role of social engagement in the inverse association between neighborhood poverty and HV.^32,35^ During childhood, peer relationships within the school environment may be critical in fostering socioemotional development.^60,61^ Factors like school connectedness, active engagement in learning, strong investment in the school community, and a positive school climate have been shown to offset risks associated with mental and emotional well-being and behavioral problems.^28,29,62,63^

While some work has investigated positive family environment (eg, supportive parenting styles) and adolescent brain structure and function,^64^ we did not find a moderating interaction of family environment in the association between neighborhood single-parent households and right HV. Previous studies found that children living in single-parent households often exhibited more deviant behaviors and engaged in risky activities, perhaps through a lack of informal parental supervision.^65,66^ A greater proportion of single-parent households may be associated with downstream outcomes in youth behavior, creating adverse social environments for other children and adolescents in the community (ie, schools). If the association of neighborhood single-parent households and HV is explained by the downstream outcomes of school-level deviant youth behavior (ie, peer victimization) among other youths, then a positive school environment may be more protective (compared with the family environment) against negative peer relationships, especially in the context of single-parent families.

### Limitations

This study has several limitations. First, given the observational, cross-sectional design of this dataset, no conclusions about causality or direction of associations among neighborhood characteristics, HV, and school environments could be established. While the moderating interaction with the school environment may suggest a protective role of a better school environment against adverse social environments, it may also be possible that children who perceived the school environment as positive were less susceptible to social stressors in the neighborhood. Future studies investigating the association of the social environment with longitudinal changes in brain structure are warranted. Second, because family and school environments were all self-report measures, these subjective perceptions could vary individually, which may not be the most accurate or reliable measurement. Third, effect sizes for associations between indices of ADI and HV were small, which is consistent with prior findings.^18,35^ This may be partly due to the limited variability in neighborhood deprivation indices given that most participants in this study came from households of middle to high socioeconomic level. Nevertheless, these findings may have a relevant and meaningful impact, particularly when applied to large populations.^67^

## Conclusions

This cross-sectional study found that a greater proportion of single-parent households in a neighborhood was associated with reduced right HV in children. In addition, our findings suggest that this association may be buffered by better school environments. Replicating these results in a longitudinal causal framework would highlight the importance of social support and family structures in communities and schools. This could have implications for advocating for policies that foster a positive school environment and social support for communities with greater proportions of single-parent households.

## Notes

Supplement 1. **eTable 1.** Summary of the number of missing participants for relevant variables **eTable 2.** Comparison of sociodemographic characteristics between included and excluded participants **eTable 3.** Variance inflation factor for bilateral HV **eTable 4.** Stepwise backward elimination for bilateral HV **eTable 5.** Sensitivity Analysis: Multivariable models for bilateral HV in QC samples (n = 9081) **eTable 6.** Sensitivity Analysis: Interaction models of neighborhood single-parent households-by-school and family environments for right HV in QC samples (n = 9081) **eFigure 1.** Comparison of all continuous sociodemographic characteristics between included and excluded participants using quantile-quantile plot **eFigure 2.** Comparison of sociodemographic characteristics between included and excluded participants **eFigure 3.** Comparison of sociodemographic individual-level and neighborhood-level characteristics between included and excluded participants **eFigure 4.** Distribution of neighborhood-level deprivation indices **eFigure 5.** Distribution of individual-level continuous variables **eFigure 6.** Box plots of bilateral HV by categorical variables **eFigure 7.** Pairwise correlation and scatter plots of neighborhood-level deprivation indices and bilateral HV **eFigure 8.** Pairwise correlation and scatter plots of individual-level continuous variables and bilateral HV **eFigure 9.** Pairwise correlation and scatter plots of family and school environments with neighborhood-level deprivation indices
eReferences.

Supplement 2.
Data Sharing Statement
