# Supplementary material for: Aspects of Area Deprivation Index in Relation to Hippocampal Volume Among Children
Source: JAMA Netw Open. 2024 Jun 12;7(6):e2416484. doi: 10.1001/jamanetworkopen.2024.16484 (PMC11170298; doi:10.1001/jamanetworkopen.2024.16484)
Supplement: Supplement 1. — eTable 1. Summary of the number of missing participants for relevant variables eTable 2. Comparison of sociodemographic characteristics between included and excluded participants eTable 3. Variance inflation factor for bilateral HV eTable 4. Stepwise backward elimination for bilateral HV eTable 5. Sensitivity Analysis: Multivariable models for bilateral HV in QC samples (n = 9081) eTable 6. Sensitivity Analysis: Interaction models of neighborhood single-parent households-by-school and family environments for right HV in QC samples (n = 9081) eFigure 1. Comparison of all continuous sociodemographic characteristics between included and excluded participants using quantile-quantile plot eFigure 2. Comparison of sociodemographic characteristics between included and excluded participants eFigure 3. Comparison of sociodemographic individual-level and neighborhood-level characteristics between included and excluded participants eFigure 4. Distribution of neighborhood-level deprivation indices eFigure 5. Distribution of individual-level continuous variables eFigure 6. Box plots of bilateral HV by categorical variables eFigure 7. Pairwise correlation and scatter plots of neighborhood-level deprivation indices and bilateral HV eFigure 8. Pairwise correlation and scatter plots of individual-level continuous variables and bilateral HV eFigure 9. Pairwise correlation and scatter plots of family and school environments with neighborhood-level deprivation indices eReferences. [file jamanetwopen-e2416484-s001.pdf]

Supplemental Online Content

Ku BS, Aberizk K, Feurer C, et al. Aspects of area deprivation index in relation to hippocampal volume among children. *JAMA Netw Open*. 2024;7(6):e2416484.
doi:10.1001/jamanetworkopen.2024.16484

eTable 1. Summary of the number of missing participants for relevant variables

eTable 2. Comparison of sociodemographic characteristics between included and excluded participants

eTable 3. Variance inflation factor for bilateral HV

eTable 4. Stepwise backward elimination for bilateral HV

eTable 5: Sensitivity Analysis: Multivariable models for bilateral HV in QC samples (n = 9081)

eTable 6. Sensitivity Analysis: Interaction models of neighborhood single-parent households-by-school and family environments for right HV in QC samples (n = 9081)

eFigure 1. Comparison of all continuous sociodemographic characteristics between included and excluded participants using quantile-quantile plot

eFigure 2. Comparison of sociodemographic characteristics between included and excluded participants

eFigure 3. Comparison of sociodemographic individual-level and neighborhood-level characteristics between included and excluded participants

eFigure 4. Distribution of neighborhood-level deprivation indices

eFigure 5. Distribution of individual-level continuous variables

eFigure 6. Box plots of bilateral HV by categorical variables

eFigure 7. Pairwise correlation and scatter plots of neighborhood-level deprivation indices and bilateral HV

eFigure 8. Pairwise correlation and scatter plots of individual-level continuous variables and bilateral HV

eFigure 9. Pairwise correlation and scatter plots of family and school environments with neighborhood-level deprivation indices

eReferences.

This supplemental material has been provided by the authors to give readers additional information about their work.

© 2024 Ku BS et al. *JAMA Network Open.*

eTable 1. Summary of the number of missing participants for relevant variables.

Variables	Missing Cases
Total combined family income	1018
Valid Addresses for Neighborhood Geo-coding	703
Brain Regions of Interests	116
Positive School Environment	25
Positive Family Environment	34

© 2024 Ku BS et al. *JAMA Network Open.*

eTable 2. Comparison of sociodemographic characteristics between included and excluded participants.

	**Included participants**	**Excluded participants due to missing data**	**Excluded (%)** [a]	**P** [b]
Sample size N	10,114	1,762	14.8	
Age (median [IQR])	9.92 [9.33, 10.48]	9.92 [9.33, 10.50]		0.71
Male (%)	5294 (52.3)	902 (51.2)	14.6	0.39
Race (%)				< .001
American Indian or Alaskan Native	50 (0.5)	12 (0.7)	19.4	
Asian	200 (2.0)	43 (2.4)	17.7	
Black	1411 (14.0)	458 (26.0)	24.5	
Native Hawaiian or Pacific Islanders	10 (0.1)	5 (0.3)	33.3	
Others [c]	525 (5.2)	171 (9.7)	24.6	
Two or more races	1263 (12.5)	204 (11.6)	13.9	
White	6655 (65.8)	869 (49.3)	11.5	
Hispanic/Latino (%)	1959 (19.4)	452 (25.7)	18.8	< .001
Parents with a high school diploma or greater (%)	8862 (87.7)	1275 (72.7)	12.6	< .001
Total combined family income (median [IQR])	8.00 [6.00, 9.00]	8.00 [5.00, 9.00]		0.002
Neighborhood-level Deprivation Index				
Percentage of high school diploma (median [IQR])	92.55 [85.10, 96.26]	87.80 [76.17, 94.28]		< .001
Median annual family income	73,333.00 [51,584.75, 97,909.50]	57,533.50 [38,873.75, 85,239.25]		< .001
Income Disparity (median [IQR])	2.01 [1.24, 2.88]	2.56 [1.52, 3.59]		< .001
Percentage of house owners (median [IQR])	71.20 [52.14, 83.86]	60.16 [38.38, 79.09]		< .001
Percentage of unemployed (median [IQR])	7.37 [4.94, 10.95]	9.51 [6.01, 14.34]		< .001
Percentage of below poverty line (median [IQR])	6.89 [3.13, 14.66]	12.24 [4.66, 23.85]		< .001
Percentage below 138% of the poverty line (median [IQR])	15.75 [8.97, 28.59]	24.34 [11.14, 40.14]		< .001
Percentage of single parents (median [IQR])	13.91 [8.74, 22.70]	19.64 [11.37, 31.93]		< .001
Percentage of no car (median [IQR])	4.79 [2.13, 10.41]	7.67 [3.38, 16.95]		< .001
Intracranial Volume (median [IQR])	1,489,639.50 [1,393,115.50, 1,588,378.25]	1,461,313.50 [1,372,385.75, 1,562,422.75]		< .001
Left Hippocampal Volume (median [IQR])	4,015.45 [3,757.40, 4,287.00]	3,930.35 [3,675.30, 4,205.18]		< .001
Right Hippocampal Volume (median [IQR])	4,137.85 [3,866.93, 4,425.38]	4,066.15 [3,784.85, 4,339.58]		< .001
Positive School Environment (median [IQR])	20.00 [18.00, 22.00]	20.00 [18.00, 22.00]		0.20
Positive Family Environment (median [IQR])	3.00 [2.60, 3.00]	3.00 [2.60, 3.00]		0.68

a. Excluded % refers to the percentage of excluded participants among each level of categorical variables.
b. X^2 tests were used for categorical variables and the Mann-Whitney U tests were used for non-normal continuous variables.
c. Others include: the race that participants reported was not included in the list, or they did not know or did not disclose their race.

© 2024 Ku BS et al. *JAMA Network Open.*

eTable 3. Variance inflation factor for bilateral HV

**Left Hippocampal Volume**	
Variable	Variance Inflation Factor
Age	1.01
Sex	1.31
Non-Hispanic White	1.31
Parental high school education	1.14
Total Combined Family Income	1.61
Intracranial Volume	1.38
Median annual family income	2.12
Percentage of high school diploma	2.51
Percentage of unemployed	1.97
Percentage of single parents	2.63
**Right Hippocampal Volume**	
Variable	VIF
Age	1.01
Sex	1.30
Non-Hispanic White	1.30
Parental high school education	1.12
Total Combined Family Income	1.60
Intracranial Volume	1.38
Median annual family income	2.14
Percentage of unemployed	1.92
Percentage of single parents	2.60

© 2024 Ku BS et al. *JAMA Network Open.*

eTable 4. Stepwise backward elimination for bilateral HV.

**Left Hippocampal Volume**																		
Variables	Before stepwise elimination			Step 1			Step 2			Step 3			Step 4			Step 5		
	β	95% CI	*p*	β	95% CI	*p*	β	95% CI	*p*	β	95% CI	*p*	β	95% CI	*p*	β	95% CI	*p*
Percentage of high school diploma	0.05	0.02 to 0.09	0.003	0.05	0.02 to 0.09	< .001	0.05	0.02 to 0.09	< .001	0.05	0.02 to 0.08	< .001	0.05	0.02 to 0.08	0.001	0.05	0.02 to 0.08	< .001
Median annual family income	0.06	0.02 to 0.09	0.003	0.06	0.02 to 0.09	< .001	0.06	0.02 to 0.09	< .001	0.05	0.01 to 0.07	< .001	0.05	0.01 to 0.09	0.006	0.04	0.01 to 0.07	0.02
Income Disparity	0.02	-0.02 to 0.06	0.35	0.02	-0.02 to 0.06	0.35	0.02	-0.02 to 0.06	0.27	0.02	-0.02 to 0.05	0.37						
Percentage of house owner	0	-0.06 to 0.01	0.21	0	-0.06 to 0.01	0.2	0	-0.06 to 0.01	0.16	0	-0.05 to 0.01	0.24	0	-0.06 to 0.01	0.08			
Percentage of unemployed	-0.1	-0.10 to -0.03	< .001	-0.1	-0.10 to -0.03	< .001	-0.1	-0.10 to -0.03	< .001	-0.1	-0.10 to -0.04	< .001	-0.1	-0.09 to -0.03	< .001	-0.1	-0.10 to -0.04	< .001
Percentage of below poverty line	0	-0.07 to 0.06	0.87															
Percentage below 138% of the poverty line	0.01	-0.07 to 0.08	0.83	<0.01	-0.05 to 0.06	0.88												

© 2024 Ku BS et al. *JAMA Network Open.*

Percentage of single parents	-0.1	-0.11 to -0.03	< .001	-0.1	-0.11 to -0.03	< .001	-0.1	-0.11 to -0.03	< .001	-0.1	-0.11 to -0.04	< .001	-0.1	-0.11 to -0.04	< .001	-0.1	-0.09 to -0.03	< .001
Percentage of no-car	0	-0.05 to 0.02	0.40	0	-0.05 to 0.02	0.39	0	-0.05 to 0.02	0.4									
Intraclass Correlation of Family Groups			0.46			0.46			0.46			0.46			0.46			0.46
Intraclass Correlation of Sites			0.02			0.02			0.02			0.02			0.02			0.02
Pseudo-R square (Fixed)			0.03			0.03			0.03			0.03			0.03			0.03
Pseudo-R square (Total)			0.49			0.49			0.49			0.49			0.49			0.49

Right Hippocampal Volume

Variables	Before stepwise elimination			Step 1			Step 2			Step 3			Step 4			Step 5			Step 6		
	β	95% CI	*p*	β	95% CI	*p*	β	95% CI	*p*	β	95% CI	*p*	β	95% CI	*p*	β	95% CI	*p*	β	95% CI	*p*
Percentage of high school diploma	0.02	-0.02 to 0.06	0.20	0.02	-0.01 to 0.06	0.20	0.02	-0.01 to 0.06	0.18	0.03	-0.01 to 0.06	0.15	0.02	-0.01 to 0.05	0.17						
Median annual family income	0.06	0.02 to 0.10	0.001	0.06	0.02 to 0.10	0.001	0.06	0.02 to 0.10	0.002	0.06	0.02 to 0.09	0	0.06	0.02 to 0.09	0.002	0.07	0.03 to 0.10	< .001	0.06	0.03 to 0.09	< .001
Income Disparity	0.01	-0.03 to 0.05	0.56	0.01	-0.03 to 0.05	0.59															
Percentage of house owner	0	-0.06 to 0.02	0.30	0	-0.05 to 0.02	0.32	0	-0.06 to 0.02	0.26	0	-0.06 to 0.01	0.23	0	-0.06 to 0.00	0.09	0	-0.06 to 0.00	0.00			

© 2024 Ku BS et al. *JAMA Network Open.*

Percentage of unemployed	-0.1	-0.11 to -0.03	< .001	-0.1	-0.10 to -0.04	< .001	-0.1	-0.10 to -0.04	< .001	-0.1	-0.10 to -0.04	< .001	-0.1	-0.10 to -0.04	< .001	-0.1	-0.10 to -0.04	< .001	-0.1	-0.10 to -0.05	< .001
Percentage of below poverty line	0	-0.08 to 0.04	0.51	0	-0.08 to 0.04	0.49	0	-0.08 to 0.04	0.49												
Percentage below 138% of the poverty line	0.02	-0.06 to 0.10	0.56	0.02	-0.05 to 0.10	0.56	0.03	-0.04 to 0.10	0.41	0.01	-0.04 to 0.07	0.62									
Percentage of single parents	-0.1	-0.12 to -0.04	< .001	-0.1	-0.12 to -0.04	< .001	-0.1	-0.12 to -0.04	< .001	-0.1	-0.12 to -0.04	< .001	-0.1	-0.11 to -0.04	< .001	-0.1	-0.12 to -0.05	< .001	-0.1	-0.10 to -0.04	< .001
Percentage of no-car	<0.01	-0.03 to 0.04	0.78																		
Intraclass Correlation of Family Groups			0.43			0.43			0.43			0.43			0.43			0.43			0.43
Intraclass Correlation of Sites			0.03			0.03			0.03			0.03			0.03			0.03			0.03
Pseudo-R square (Fixed)			0.03			0.03			0.03			0.03			0.03			0.03			0.03
Pseudo-R square (Total)			0.47			0.47			0.47			0.47			0.47			0.47			0.47

Note: Nine indices of ADI were entered into the model with family and site as random intercepts. For each step, the index with the highest p-value was removed until all remaining indices' p-values were less than 0.05 according to the standard significance level. [1]

© 2024 Ku BS et al. *JAMA Network Open.*

eTable 5: Sensitivity Analysis: Multivariable models for bilateral HV in QC samples (*n* = 9081).

**Left Hippocampal Volume**												
Variables	Model A. Before stepwise elimination				Model B. After Stepwise elimination				Model C. Adjusted for 6-individual level covariates			
	β	SE	95% CI	*p*	β	SE	95% CI	*p*	β	SE	95% CI	*p*
Age									0.02	0.01	0.01 to 0.03	0.01
Male sex									0.09	0.02	0.05 to 0.12	< .001
White non-Hispanic									0.10	0.02	0.06 to 0.14	< .001
Parental high school education									0.03	0.04	-0.06 to 0.11	0.56
Total Combined Family Income									0.02	0.00	0.01 to 0.03	< .001
Intracranial Volume									0.66	0.01	0.64 to 0.68	< .001
Neighborhood-Level Deprivation Index												
Percentage of high school diploma	0.05	0.02	0.02 to 0.09	0.005	0.05	0.02	0.02 to 0.09	0.001	-0.01	0.01	-0.04 to 0.01	0.26
Median annual family income	0.05	0.02	0.02 to 0.09	0.006	0.04	0.02	0.00 to 0.07	0.03	<0.01	0.01	-0.02 to 0.03	0.73
Income Disparity	0.02	0.02	-0.02 to 0.07	0.29								
Percentage of house owner	-0.02	0.02	-0.06 to 0.01	0.21								
Percentage of unemployed	-0.06	0.02	-0.10 to -0.03	< .001	-0.06	0.02	-0.09 to -0.04	< .001	-0.01	0.01	-0.03 to 0.01	0.35
Percentage below the poverty line	-0.01	0.03	-0.07 to 0.06	0.87								

© 2024 Ku BS et al. *JAMA Network Open.*

Percentage below 138% of the poverty line	<-0.01	0.04	-0.08 to 0.07	0.96								
Percentage of single parents	-0.07	0.02	-0.11 to -0.03	0.001	-0.06	0.02	-0.09 to -0.03	0.001	-0.02	0.01	-0.05 to 0.01	0.12
Percentage without car	-0.01	0.02	-0.03 to 0.04	0.43								
Intraclass Correlation of Family Groups				0.46				0.46				0.41
Intraclass Correlation of Sites				0.02				0.02				0.03
Pseudo-R square (Fixed)				0.03				0.03				0.47
Pseudo-R square (Total)				0.50				0.50				0.71

Right Hippocampal Volume

Variables	Model A. Before stepwise elimination				Model B. After Stepwise elimination				Model C. Adjusted for 6-individual level covariates			
	β	SE	95% CI	*p*	β	SE	95% CI	*p*	β	SE	95% CI	*p*
Age									0.01	0.01	0.00 to 0.03	0.15
Male sex									0.07	0.02	0.04 to 0.10	< .001
White non-Hispanic									0.05	0.02	0.01 to 0.09	0.013
Parental high school education									-0.03	0.04	-0.12 to 0.05	0.47
Total combined family income									0.02	0.00	0.01 to 0.03	< .001
Intracranial volume									0.63	0.01	0.61 to 0.65	< .001
Neighborhood-Level Deprivation Index												
Percentage of high school diploma	0.02	0.02	-0.02 to 0.06	0.26								

© 2024 Ku BS et al. *JAMA Network Open.*

Median annual family income	0.05	0.02	0.02 to 0.09	0.005	0.05	0.02	0.02 to 0.08	0.001	<-0.01	0.01	-0.03 to 0.02	0.74
Income Disparity	0.02	0.02	-0.03 to 0.06	0.45								
Percentage of house owner	-0.02	0.02	-0.06 to 0.02	0.31								
Percentage of unemployed	-0.07	0.02	-0.10 to -0.03	< .001	-0.07	0.02	-0.10 to -0.04	< .001	-0.01	0.01	-0.04 to 0.01	0.25
Percentage below the poverty line	-0.02	0.03	-0.08 to 0.04	0.47								
Percentage below 138% of the poverty line	0.01	0.04	-0.06 to 0.09	0.79								
Percentage of single parents	-0.07	0.02	-0.12 to -0.03	< .001	-0.07	0.02	-0.11 to -0.04	< .001	-0.03	0.01	-0.06 to -0.01	0.02
Percentage without car	0.00	0.02	-0.03 to 0.04	0.84								
Intraclass Correlation of Family Groups				0.43				0.43				0.38
Intraclass Correlation of Sites				0.03				0.03				0.02
Pseudo-R square (Fixed)				0.03				0.03				0.43
Pseudo-R square (Total)				0.47				0.47				0.66

Note: In the sensitivity analysis for quality control (QC), we included only those who met the criteria for "T1w data recommended for inclusion," which was a subset of those who passed QC for Freesurfer and T1 series and did not have missing QC data (imgincl_t1w_include=1) (n = 9081).[2,3]

© 2024 Ku BS et al. *JAMA Network Open.*

eTable 6. Sensitivity Analysis: Interaction models of neighborhood single-parent households-by-school and family environments for right HV in QC samples (n = 9081).

	Interaction Model with Family Environment				Interaction Model with School Environment			
	β	SE	95% CI	*p*	β	SE	95% CI	*p*
Age	0.01	0.01	-0.01 to 0.04	0.16	0.01	0.01	0.00 to 0.03	0.15
Male sex	0.07	0.02	0.04 to 0.11	< .001	0.07	0.02	0.04 to 0.11	< .001
White non-Hispanic	0.05	0.02	0.01 to 0.09	0.01	0.05	0.02	0.01 to 0.09	0.01
Parental high school education	-0.03	0.04	-0.12 to 0.05	0.47	-0.03	0.04	-0.11 to 0.06	0.52
Total combined family income	0.02	<0.01	0.01 to 0.03	< .001	0.02	0.00	0.01 to 0.03	< .001
Intracranial volume	0.63	0.01	0.61 to 0.65	< .001	0.63	0.01	0.61 to 0.65	< .001
Median annual family income	0.00	0.01	-0.02 to 0.03	0.73	0.00	0.01	-0.03 to 0.02	0.75
Percentage of unemployed	-0.01	0.01	-0.04 to 0.01	0.25	-0.01	0.01	-0.04 to 0.01	0.29
Percentage of single parents	-0.03	0.01	-0.06 to 0.00	0.01	-0.03	0.01	-0.06 to -0.01	0.01
Positive family environment	0.01	0.01	-0.01 to 0.02	0.39				
Percentage of single-parent households-by-family environment	<0.01	0.01	-0.02 to 0.01	0.65				
Positive school environment					0.01	0.01	-0.01 to 0.02	0.21
Percentage of single-parent households-by-school environment					0.02	0.01	0.01 to 0.03	0.01
Intraclass Correlation of Family Groups				0.38				0.38
Intraclass Correlation of Sites				0.02				0.02
Pseudo-R square (Total)				0.66				0.66

Note: In the sensitivity analysis for quality control (QC), we included only those who met the criteria for “T1w data recommended for inclusion,” which was a subset of those who passed QC for Freesurfer and T1 series and did not have missing QC data (imgincl_t1w_include=1) (n = 9081).[2,3]

© 2024 Ku BS et al. *JAMA Network Open.*

eFigure 1. Comparison of all continuous sociodemographic characteristics between included and excluded participants using quantile-quantile plot.

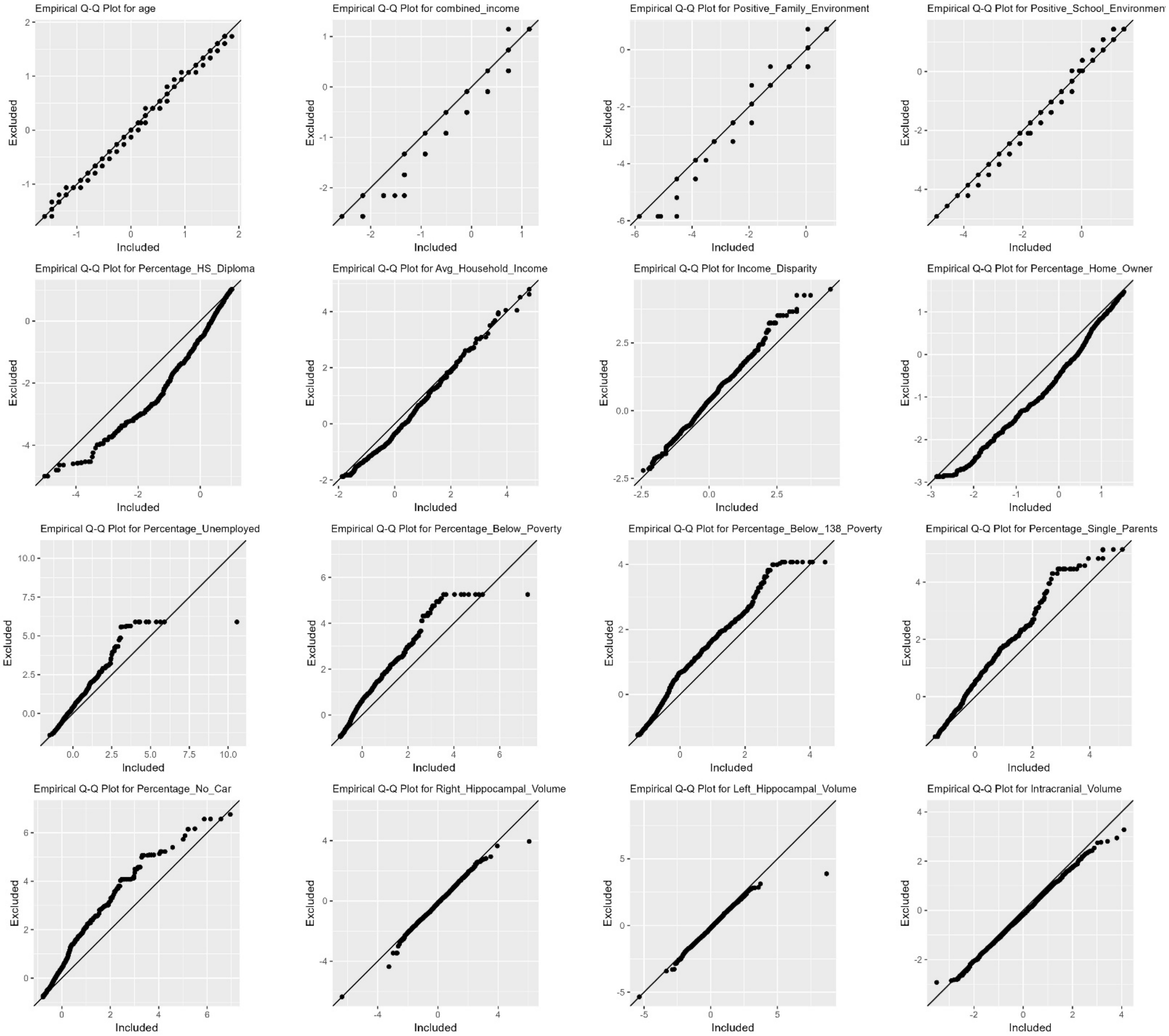

Note: Empirical quantile-quantile (Q-Q) plots compare the distributions of all continuous variables between the included (N =10114) and excluded (N = 1762) participants. Each plot displays the quantiles of the included group (x-axis) and excluded group (y-axis), with the 45-degree reference line representing identical distributions. Points closely following the reference line indicate similar distributions, while deviations suggest distributional differences between groups. Points that fall above the reference line indicate that the corresponding quantiles in the excluded dataset are higher than those in the included dataset, while points below the line suggest the opposite.

© 2024 Ku BS et al. *JAMA Network Open.*

eFigure 2. Comparison of sociodemographic characteristics between included and excluded participants.

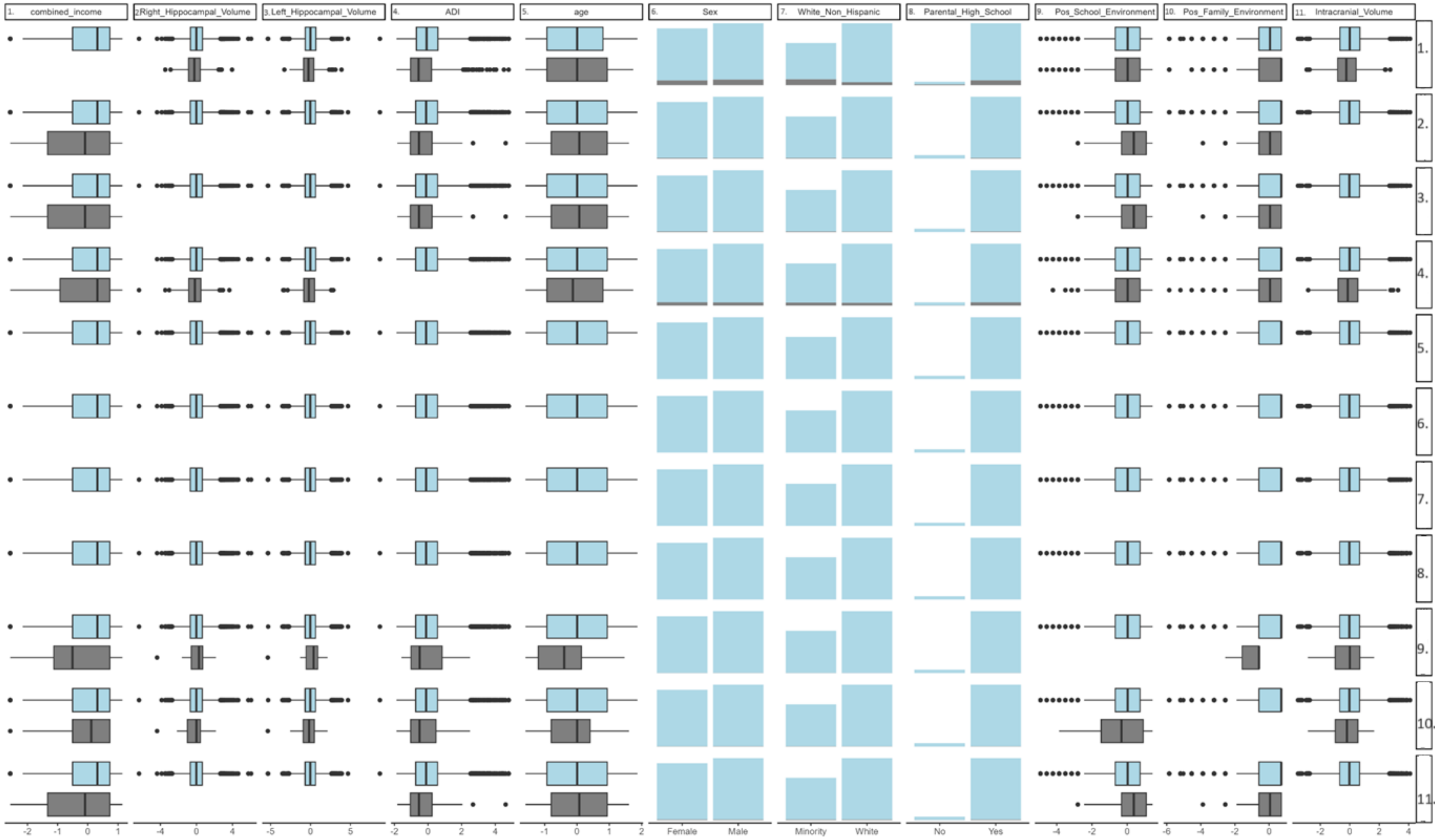

Abbreviations: ADI, area-level deprivation index; Parental_high_school, whether at least one of the parents has obtained a high school diploma.

Note: The GGally() and finalfit() packages were used to determine whether the missingness of key predictor variables (total combined family income and ADI) was related to the missingness of demographic variables and whether the missingness of outcome variables (right and left hippocampal volume) were related to the ADI and hippocampal volume. Box plots compare the distribution of included (blue) and excluded (gray) participants across variables.

© 2024 Ku BS et al. *JAMA Network Open.*

eFigure 3. Comparison of sociodemographic individual-level and neighborhood-level characteristics between included and excluded participants.

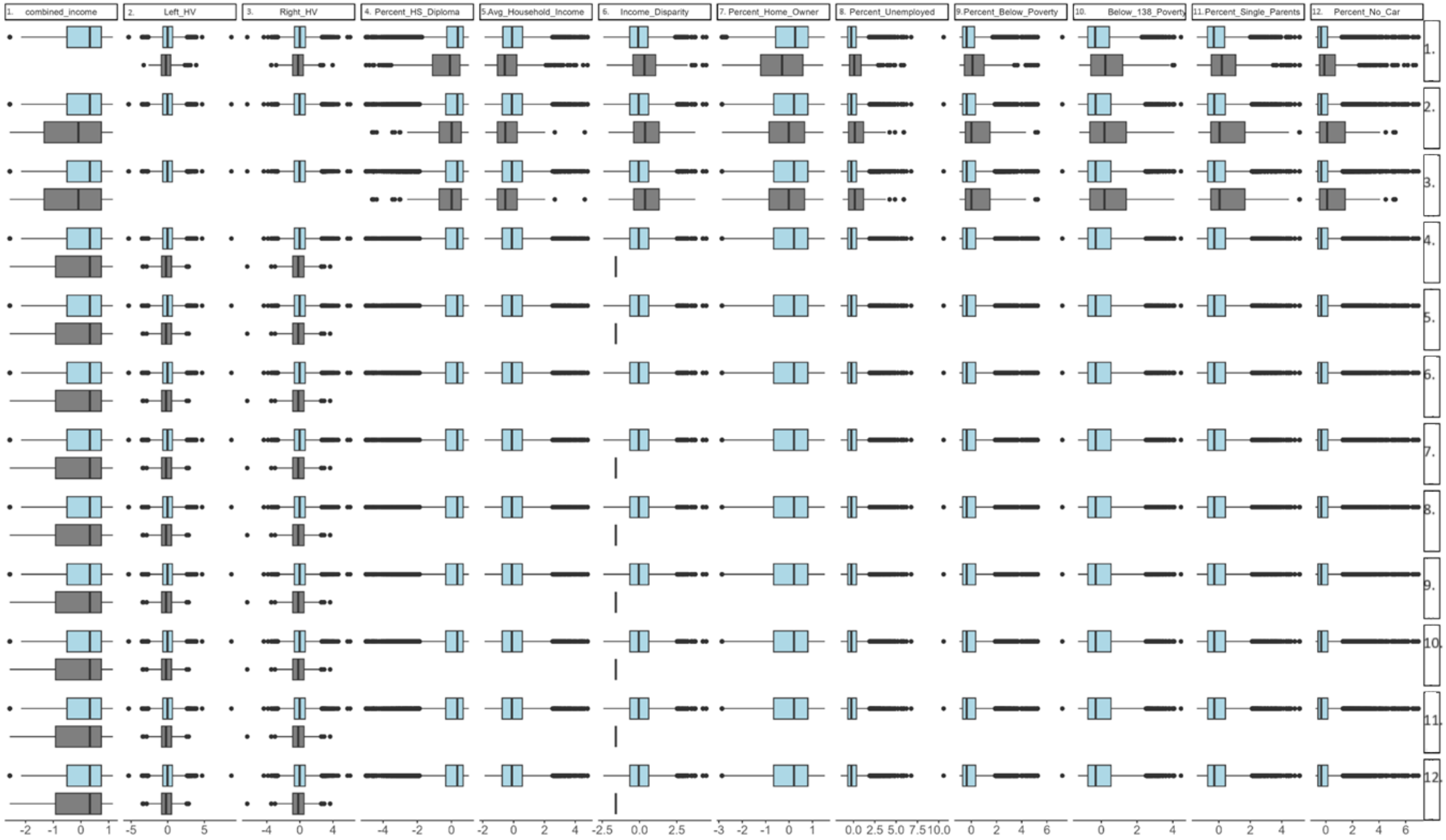

Abbreviations: combined income, total combined family income; HV, hippocampal volume; HS, high school.

Note: The GGally() and finalfit() packages were used to determine whether the missingness of total combined family income was related to the missingness of nine neighborhood-level deprivation indices. Box plots compare the distribution of included (blue) and excluded (gray) participants across variables.

© 2024 Ku BS et al. *JAMA Network Open.*

eFigure 4. Distribution of neighborhood-level deprivation indices.

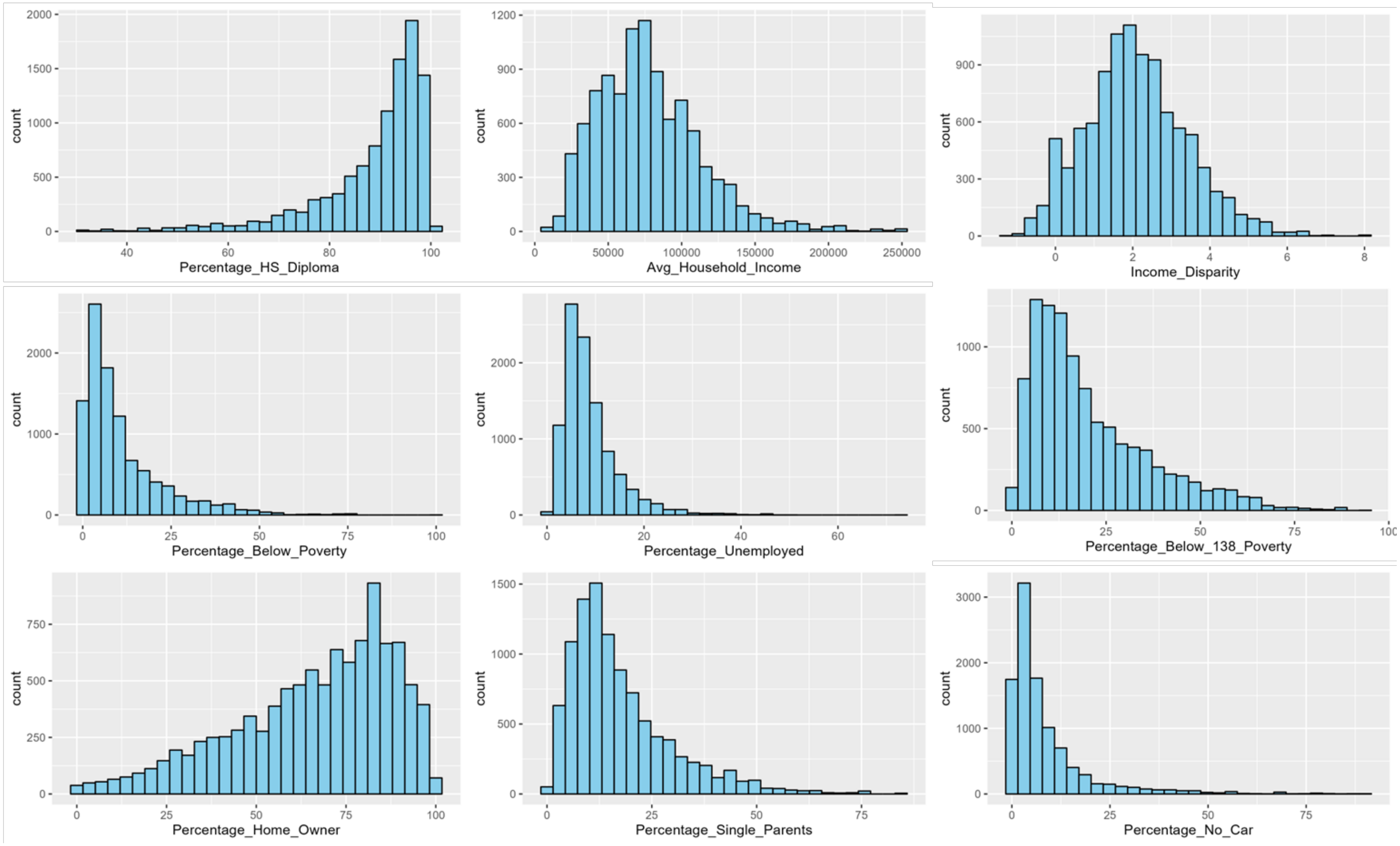

© 2024 Ku BS et al. *JAMA Network Open.*

eFigure 5. Distribution of individual-level continuous variables.

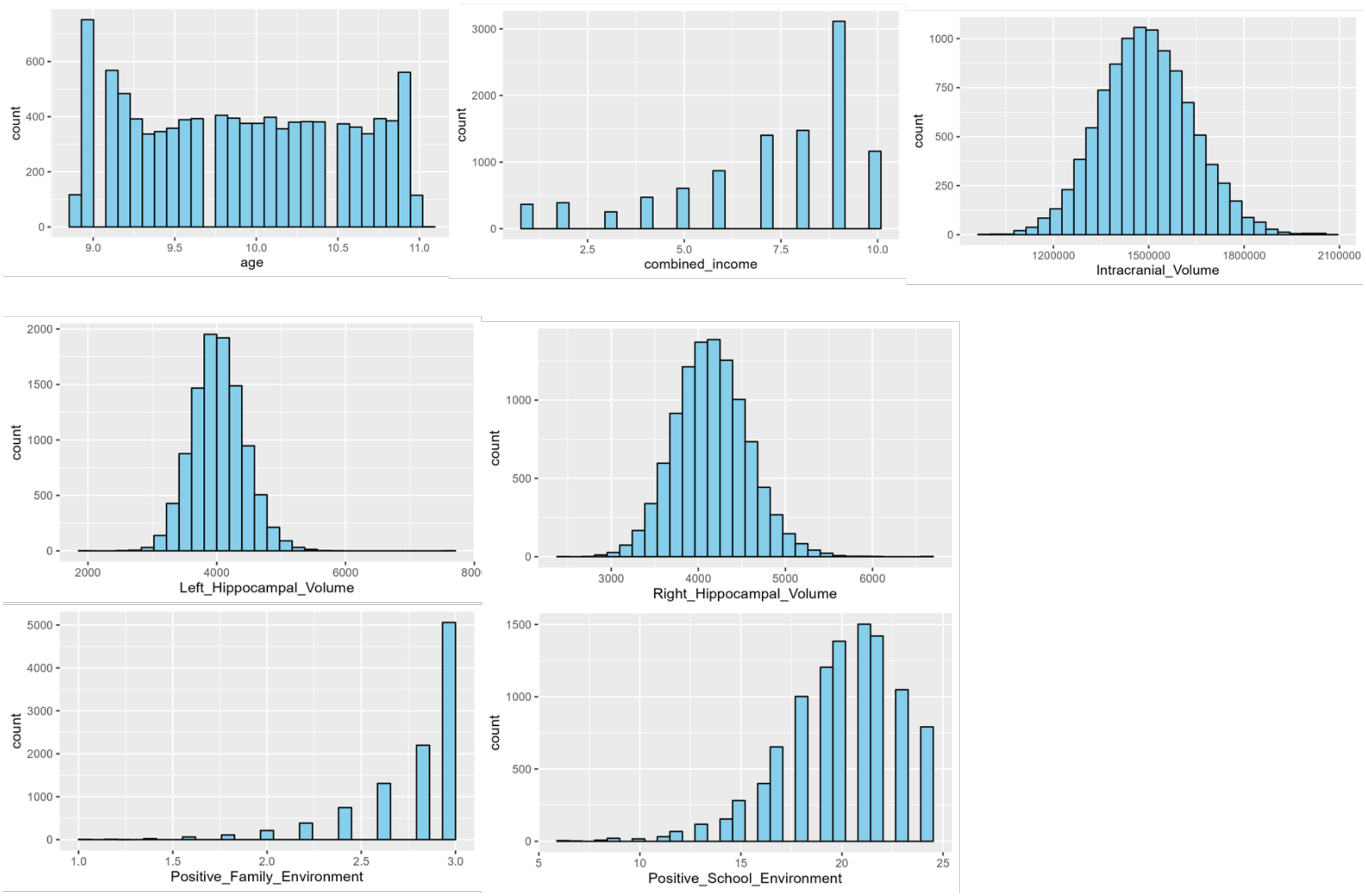

© 2024 Ku BS et al. *JAMA Network Open.*

eFigure 6. Boxplots of bilateral HV by categorical variables.

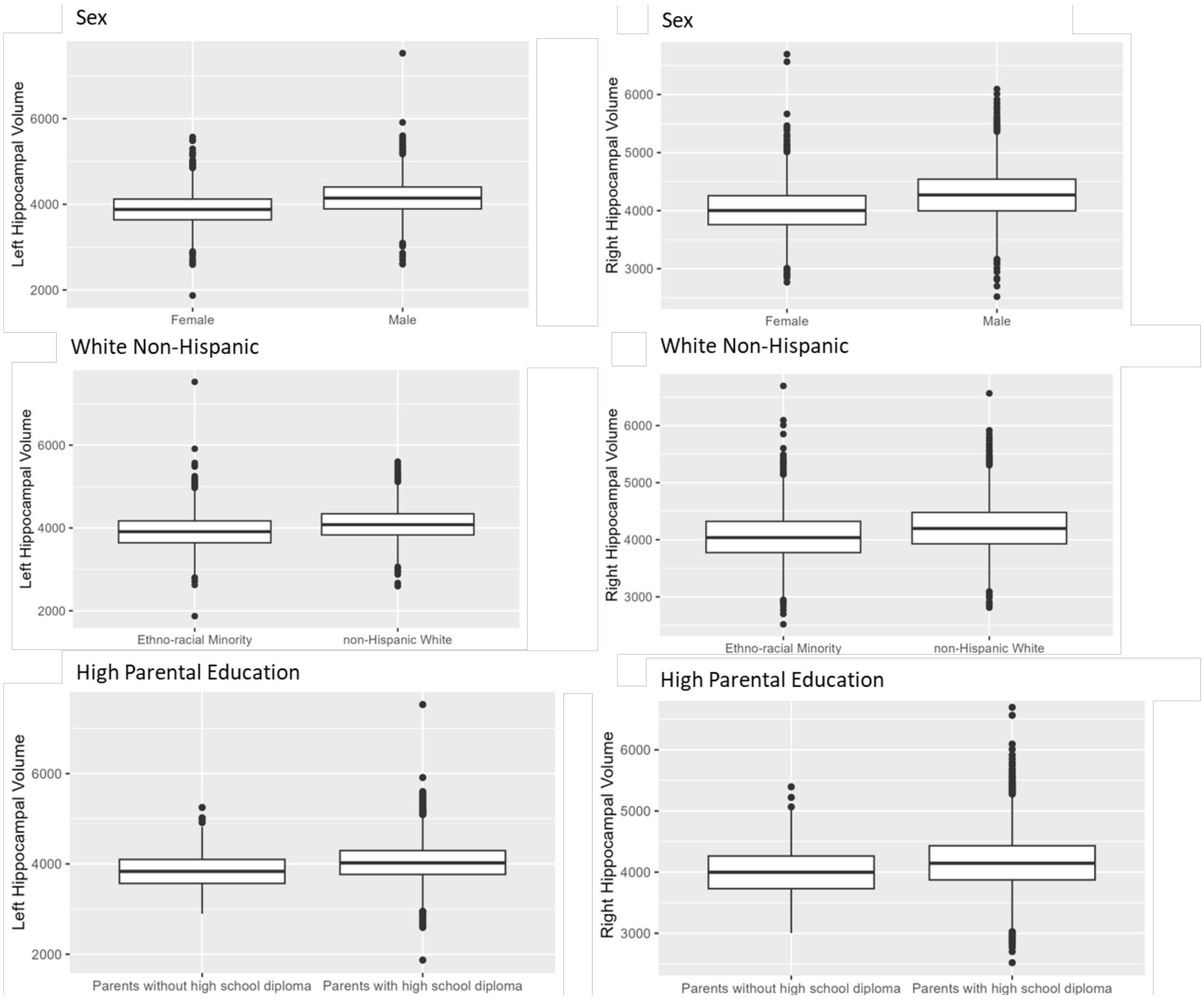

© 2024 Ku BS et al. *JAMA Network Open.*

eFigure 7. Pairwise correlation and scatter plots of neighborhood-level deprivation indices and bilateral HV.

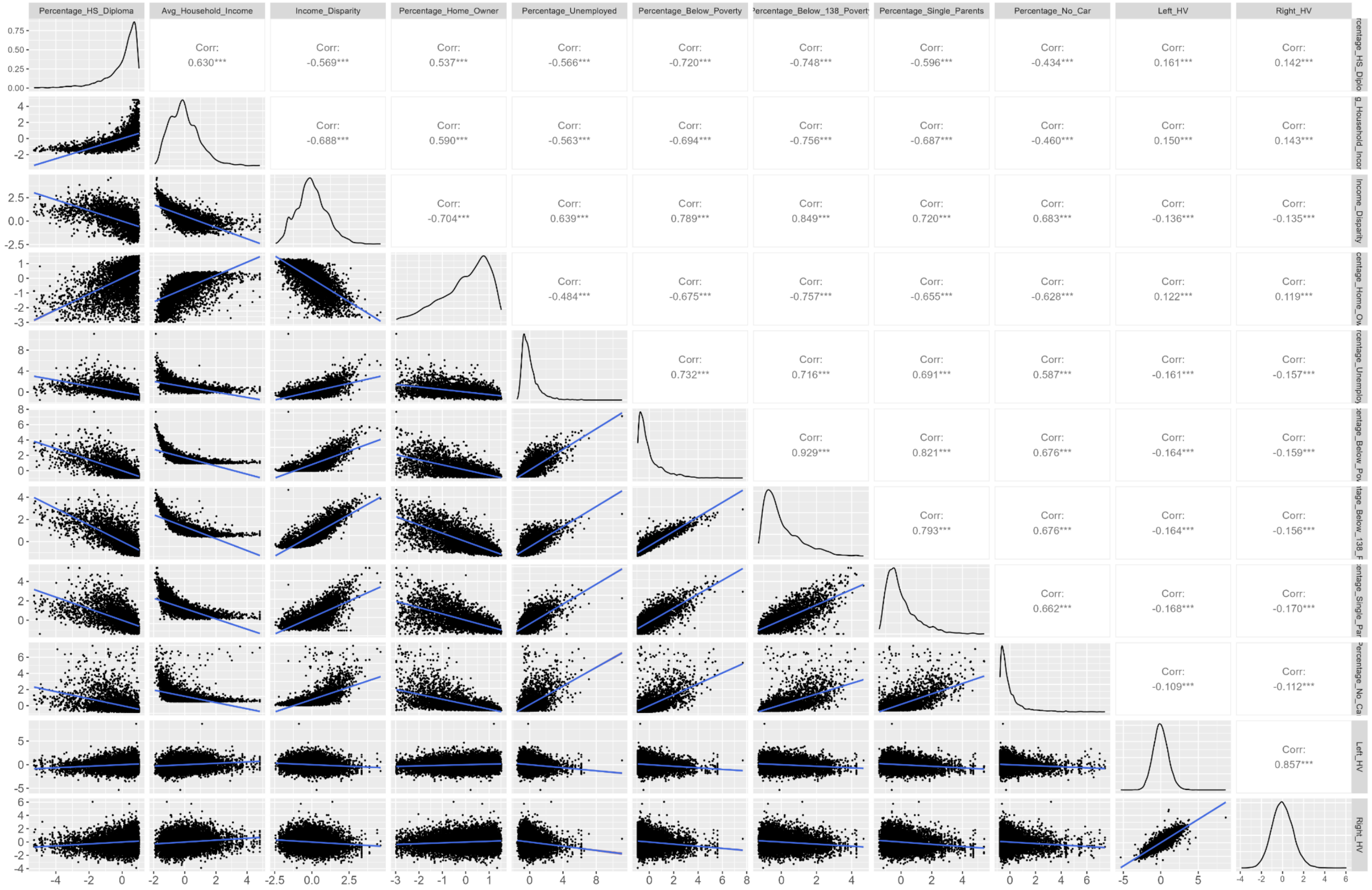

Abbreviations: HS, high school; Avg, Average; HV, hippocampal volume.

Note: Pair-wise plots of the neighborhood-level deprivation variables and hippocampal volumes were compared using Pearson correlation performed with *ggpairs* function from the "GGally" package. Individual variable distributions on the main diagonal. The lower triangle presents the scatterplots. The blue lines on scatterplots reflect the line of best fit. The upper triangle presents the Pearson correlation. The asterisks indicated a statistically significant correlation between variables by a two-tailed p-value (* p<0.05, ** p<0.01, *** p<0.001).

© 2024 Ku BS et al. *JAMA Network Open.*

eFigure 8. Pairwise correlation and scatter plots of individual-level continuous variables and bilateral HV.

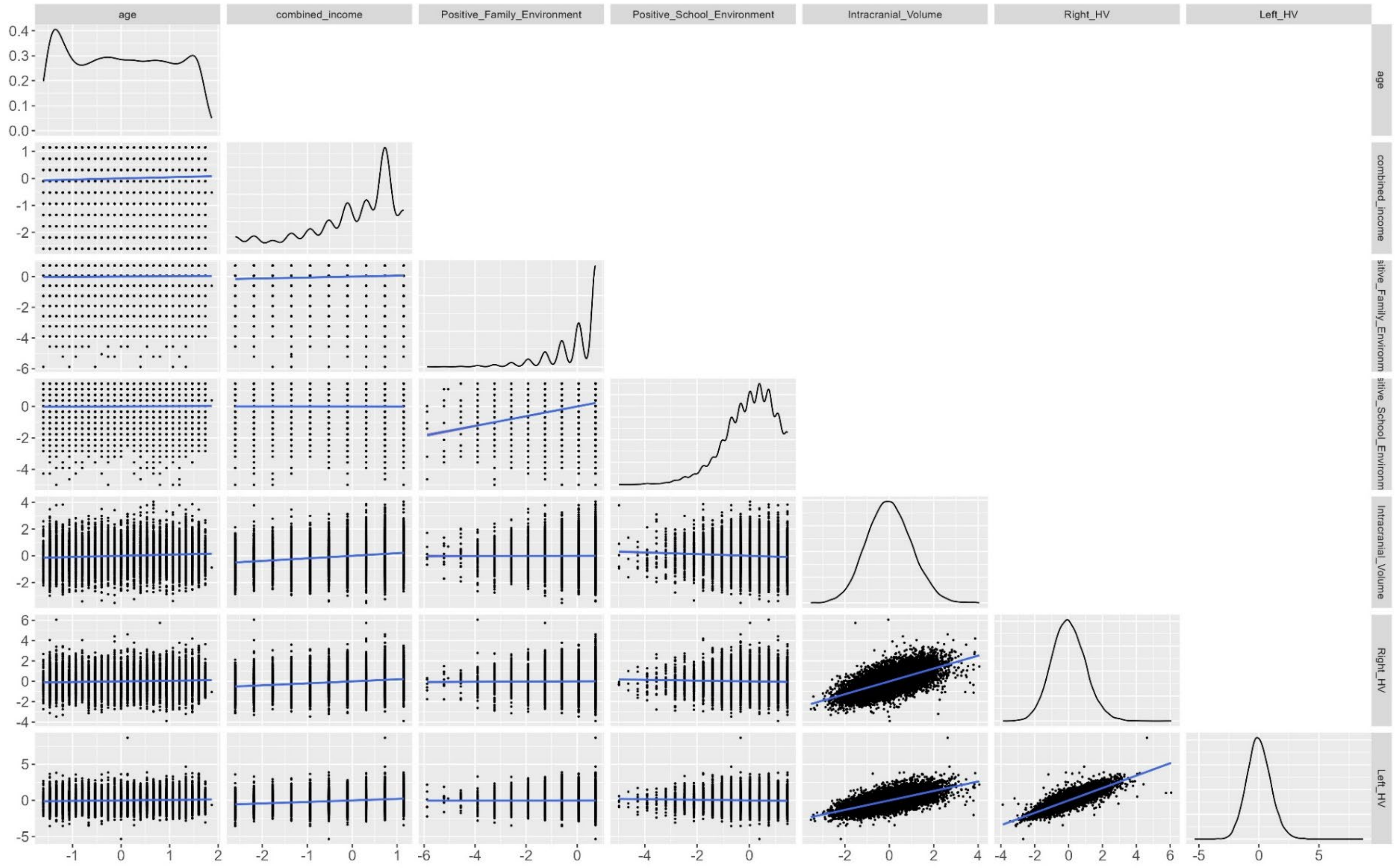

Abbreviations: combined income, combined family household income; HV, hippocampal volume.

Note: Pair-wise plots of the continuous demographic variables and hippocampal volumes were compared using Pearson correlation performed with *ggpairs* function from the “GGally” package. Individual variable distributions on the main diagonal. The lower triangle presents the scatterplots. The blue lines on scatterplots reflect the line of best fit.

© 2024 Ku BS et al. *JAMA Network Open.*

eFigure 9. Pairwise correlation and scatter plots of family and school environments and neighborhood-level deprivation indices.

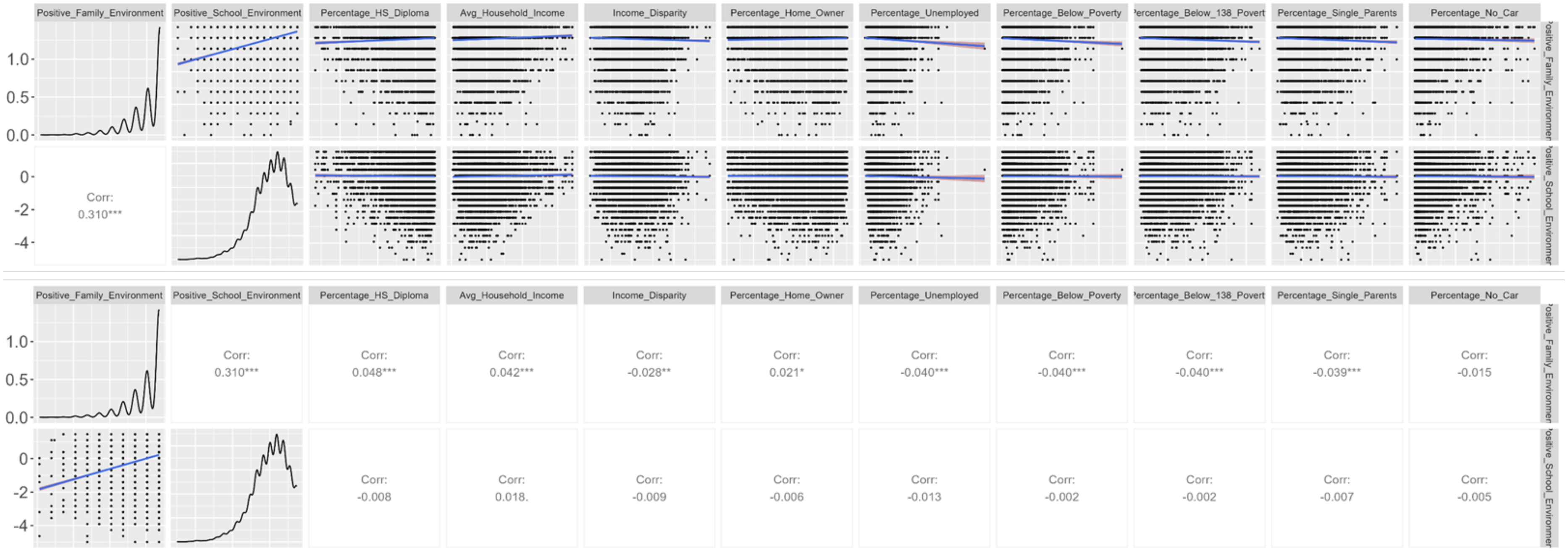

Abbreviations: HS, high school; Avg, Average.

Note: Pair-wise plots of the Neighborhood Deprivation Indices and family and school environment were compared using Pearson correlation performed with *ggpairs* function from the "GGally" package. Individual variable distributions on the main diagonal. The first two rows upper right present the scatterplots. The blue lines on scatterplots reflect the line of best fit. The last two rows upper right present the Pearson correlation. The pairwise correlation between Neighborhood Deprivation Indices was cut out since it has been presented in eFigure 4. The asterisks indicated a statistically significant correlation between variables by a two-tailed p-value (* p<0.05, ** p<0.01, *** p<0.001).

© 2024 Ku BS et al. *JAMA Network Open.*

Table for Abbreviations:

ADI: Area-level Deprivation Indices

HS: High School

HV: Hippocampal Volume

IQR: Interquartile Range

QC: Quality Control

© 2024 Ku BS et al. *JAMA Network Open.*

eReferences

1. Steyerberg EW. Clinical Prediction Models. *Statistics for Biology and Health*. 2019:211. doi:10.1007/978-3-030-16399-0
2. Hagler DJ, Jr., Hatton S, Cornejo MD, et al. Image processing and analysis methods for the Adolescent Brain Cognitive Development Study. *Neuroimage*. Nov 15 2019;202:116091. doi:10.1016/j.neuroimage.2019.116091
3. Saragosa-Harris NM, Chaku N, MacSweeney N, et al. A practical guide for researchers and reviewers using the ABCD Study and other large longitudinal datasets. *Developmental Cognitive Neuroscience*. 2022/06/01/ 2022;55:101115. doi:https://doi.org/10.1016/j.dcn.2022.101115

© 2024 Ku BS et al. *JAMA Network Open.*
