# Supplementary material for: Aspects of Area Deprivation Index in Relation to Hippocampal Volume Among Children
Source: JAMA Netw Open. 2024 Jun 12;7(6):e2416484. doi: 10.1001/jamanetworkopen.2024.16484 (PMC11170298; doi:10.1001/jamanetworkopen.2024.16484)
Supplement: Supplement 2. — Data Sharing Statement [file jamanetwopen-e2416484-s002.pdf]

Data Sharing Statement

Ku. Aspects of Area Deprivation Index in Relation to Hippocampal Volume Among Children. *JAMA Netw Open*. Published online June 12, 2024. doi:10.1001/jamanetworkopen.2024.16484

Data
Data available: Yes
Data types: Other (please specify)
Additional Information: The Adolescent Brain and Cognitive Development (ABCD) study anonymized data are released annually and are publicly available via the National Institute of Mental Health Data Archive (NDA).
How to access data: Guidance on accessing ABCD data can be found on the ABCD study data-sharing webpage: https://abcdstudy.org/scientists_data_sharing.html . The ABCD data repository grows and changes over time. All data are made available from the ABCD study (https://nda.nih.gov/abcd/request-access) to researchers from universities and other institutions who submit research inquiries and are approved following institutional review board and NDA approval.
When available: With publication

Supporting Documents
Document types: Statistical/analytic code
How to access documents: In-house R scripts and outputs for all analyses can be found in the GitHub repository at https://github.com/Emorod/ABCD-ADI_HV .
When available: With publication

Additional Information
Who can access the data: researchers whose proposed use of the data has been approved
Types of analyses: for a specified purpose
Mechanisms of data availability: All data are made available from the ABCD study (https://nda.nih.gov/abcd/request-access) to researchers from universities and other institutions who submit research inquiries and are approved following institutional review board and NDA approval.
